# Photodynamic therapy for infected foot ulcers in people with diabetes mellitus: a systematic review

**DOI:** 10.1590/1516-3180.2022.0476.27022023

**Published:** 2023-05-12

**Authors:** Maria Girlane Sousa Albuquerque Brandão, Maria Aline Moreira Ximenes, Danilo Ferreira de Sousa, Vivian Saraiva Veras, Lívia Moreira Barros, Soraia Assad Nasbine Rabeh, Idevania Geraldina Costa, Thiago Moura de Araújo

**Affiliations:** I Doctoral Student, Nursing School, Universidade de São Paulo (USP), Ribeirão Preto (SP), Brazil.; II Doctoral Student, Department of Nursing, Universidade Federal do Ceará (UFC), Fortaleza (CE), Brazil.; III Doctoral Student, Department of Nursing, Universidade Federal do Ceará (UFC), Fortaleza (CE), Brazil.; IV PhD. Professor, Department of Nursing, Universidade da Integração Internacional da Lusofonia Afro-Brasileira, Redenção (CE), Brazil.; V PhD. Professor, Department of Nursing, Universidade da Integração Internacional da Lusofonia Afro-Brasileira, Redenção (CE), Brazil.; VI PhD. Professor, Department of Nursing, Universidade de São Paulo (USP), Ribeirão Preto (SP), Brazil.; VII PhD. Professor, Department of Nursing, Lakehead University, Canada.; VIII PhD. Professor, Department of Nursing, Health Sciences Institute, Universidade da Integração Internacional da Lusofonia Afro-Brasileira, Redenção (CE), Brazil.

**Keywords:** Photochemotherapy, Oxidative stress, Infections, Diabetic foot, Laser therapy, Laser therapies, Phototherapies, Foot ulcers

## Abstract

**BACKGROUND:**

Ulceration of the feet in patients with diabetes is a frequent complication that increases morbidity, mortality, hospitalization, treatment costs, and non-traumatic amputations.

**OBJECTIVE:**

To present a systematic review of the treatment of patients with diabetes mellitus and infected foot ulcers using photodynamic therapy.

**DESIGN AND SETTING:**

A systematic review was performed in the postgraduate program in nursing at the Universidade da Integração Internacional da Lusofonia Afro-Brasileira, Ceará, Brazil.

**METHODS:**

PubMed, CINAHL, Web of Science, EMBASE, Cochrane Library, Scopus, and LILACS databases were screened. The methodological quality, risk of bias, and quality of evidence of each study were assessed. Review Manager was used for the meta-analysis.

**RESULTS:**

Four studies were included. They highlighted significantly better outcomes in patient groups treated with photodynamic therapy than those in the control groups that were treated with topical collagenase and chloramphenicol (P = 0.036), absorbent (P < 0.001), or dry covers (P = 0.002). Significant improvements were noted in terms of the microbial load in the ulcers and tissue repair, with a reported reduction in the need for amputation by up to 35 times. Photodynamic therapy resulted in significantly better outcomes between the experimental and control groups (P = 0.04).

**CONCLUSION:**

Photodynamic therapy is significantly more effective in treating infected foot ulcers than standard therapies.

**SYSTEMATIC REVIEW REGISTRATION:**

International Prospective Register of Systematic Reviews (PROSPERO) - CRD42020214187, https://www.crd.york.ac.uk/prospero/display_record.php?RecordID=214187.

## INTRODUCTION

Ulcers in the feet of patients with diabetes mellitus (DM) are a frequent complication and a relevant cause of morbidity and mortality, increased rates of hospitalization and higher treatment costs, and non-traumatic amputations of lower limbs.^1^ Approximately 60% of people who undergo amputations do so for infected ulcers in the feet.^2^

Infection is one of the leading causes of amputations in patients with DM; due to delayed healing in DM, infections promote exudate, swelling, microbial growth, friability, and hemorrhagic granulation tissue.^3,4^ Moreover, when not treated correctly, it increases the risk of osteomyelitis or sepsis.^5^ Therefore, antibiotic therapy should be implemented immediately after identifying the infection in the wound bed to avoid severe complications, such as amputation.

The standard treatment for diabetic foot ulcers (DFU) generally includes cleaning and removal of the necrotic tissue, improving blood circulation, maintenance of a moist environment, and infection control.^6^ However, the classic topical therapies for DFU are costly and include low efficacy in the presence of multidrug-resistant bacteria.^4,7^ Additionally, topical therapies are cytotoxic and delay healing.^8^ Therefore, for DFU, the standard treatment alone is not sufficient for adequate healing and prevention of infections. This fact highlights the relevance of implementing new adjuvant therapies in the treatment of complex ulcers, such as DFU.^3,9^

Several studies have suggested photodynamic therapy as a viable option for treating infections in the treatment and healing of DFU.^9–11^ Photodynamic therapy (PDT) involves the topical application of photosensitizers followed by illumination with Light Amplification by Stimulated Emission of Radiation (LASERs) or Light Emitter Diode (LED) light, which along with tissue oxygen induces the formation of reactive oxygen species and a high local cytotoxic effect, thus fighting the local infection.^12^

Systematic reviews, meta-analyses, and integrative and narrative reviews have been published regarding the effects of DFU in animal, human, and *in vitro* studies. These studies considered wounds of several etiologies. However, to date, there is no systematic review and meta-analysis concerning the effectiveness of PDT in DFU.

Although there are favorable reports regarding the application of PDT,^11,12^ newer investigations can provide additional evidence about the effectiveness of PDT in the treatment of DFU.^13^ A study identified that health professionals, even those with qualifications to operate LASERs, have doubts regarding the dosage, wavelength, time, and number of applications to be used.^14^ Therefore, it is important that health professionals, especially wound experts, understand the new approach of PDT in treating infected ulcers and incorporate it into their practice to promote optimized healing and reduce the number of complications due to DFU.^1,15^

The study is relevant in combining the effects and protocols of DFU as an adjuvant therapy in infection reduction, optimizing DFU healing, and offering scientific evidence to reduce bacterial resistance and amputations.

## OBJECTIVE

This study aimed to provide a systematic review and meta-analysis of the effectiveness of PDT in the treatment of infected DFU.

## METHODS

### Protocol and registry

This systematic review was performed according to the guidelines of the Joanna Briggs Institute (JBI) and the Preferred Reporting Items for Systematic reviews and Meta-analyses (PRISMA).^16,17^ It was registered in PROSPERO (CRD42020214187) https://www.crd.york.ac.uk/prospero/display_record.php?RecordID=214187.

### Focal issue

The research question “What is the effectiveness of PDT in the reduction of infection and healing process of infected foot ulcers in patients with DM?” was formulated using the acronym population, intervention, comparison, results, and studies (PICOS).

### Study selection

Clinical trials without limitations on the time of publication or language were included if their data were completely available. The following studies were excluded: incomplete studies in annals of events, studies in animal or *in vitro* models, and studies with ulcers secondary to injuries of other etiologies.

### Research strategy and search for scientific evidence

The search was performed between August and December 2020 and reviewed in June 2022. The databases of PubMed, CINAHL, Web of Science, EMBASE, Cochrane Library, Scopus, and LILACS were searched using descriptors, entry terms, and keywords in association with the Boolean operators “AND” and “OR” (Table 1).

**Table 1:** Search terms used for each database

Databases	Search strategy
**PubMed, CINAHL, Web of Science e Scopus**	(“Photochemotherapy” [MeSH] OR “Photochemotherapy” [All fields] OR “Photodynamic therapy” OR “PDT”) AND (“Diabetic Foot” [MeSH] OR “Diabetic Foot” [All Fields] OR “Foot Ulcer”)
**EMBASE**	(“Photochemotherapy” OR “Photodynamic therapy” OR “PDT”) AND (“diabetic foot” OR “foot ulcer”)
**Cochrane Library**	(“Photochemotherapy” OR “Photochemotherapy” OR “Photodynamic therapy” OR “PDT”) AND (“Diabetic Foot” OR “Diabetic Foot” OR “Foot Ulcer”)
**LILACS**	*(Fotoquimioterapia OR “Terapia Fotodinâmica”) AND (“Pé diabético”)*

### Screening and selection of studies

The screening and selection of studies were performed independently by two reviewers using Rayyan software (Qatar Foundation, Qatar).^18^ The complete texts of the selected publications were analyzed by the two reviewers. They evaluated the methodological rigor^19^ and capacity to answer the research question. Disagreements were resolved by a third reviewer.

### Data extraction

An instrument for data extraction was developed by the authors for the following factors: identification, methodological attributes, PDT protocol, results, limitations, and article recommendations.

### Evaluation of the risk of bias, methodological quality, and evidence quality

The risk of bias was graded as low risk of bias, high risk of bias, or uncertain risk of bias using the Cochrane Review Manager v5.4 (Cochrane; London, United Kingdom).^20^ The methodological quality was evaluated using a checklist for randomized controlled trials.^19^ The evidence quality was evaluated as high, moderate, low, or very low using GRADEpro GDT https://www.gradepro.org/ (Evidence Prime, Kraków, Poland).

### Data overview

The extracted data were organized in tables. Review Manager as used for the meta-analyses using a randomized effect model and different averages. Heterogeneity was evaluated statistically using the I^2^ test. The meta-analysis was conducted using forest plots.

## RESULTS

### Description of the included studies

Four of the 76 studies identified were included in the analysis. Other studies only included ulcers of other etiologies or deviated from the eligibility criteria of this meta-analysis (Flow chart 1). Two evaluated studies did not perform real randomization or hidden allocation. In three cases, blinding was not clear (Table 2). In the risk evaluation of bias, the distribution was mainly classified as low-risk (Graphic 1).

**Flow chart:**
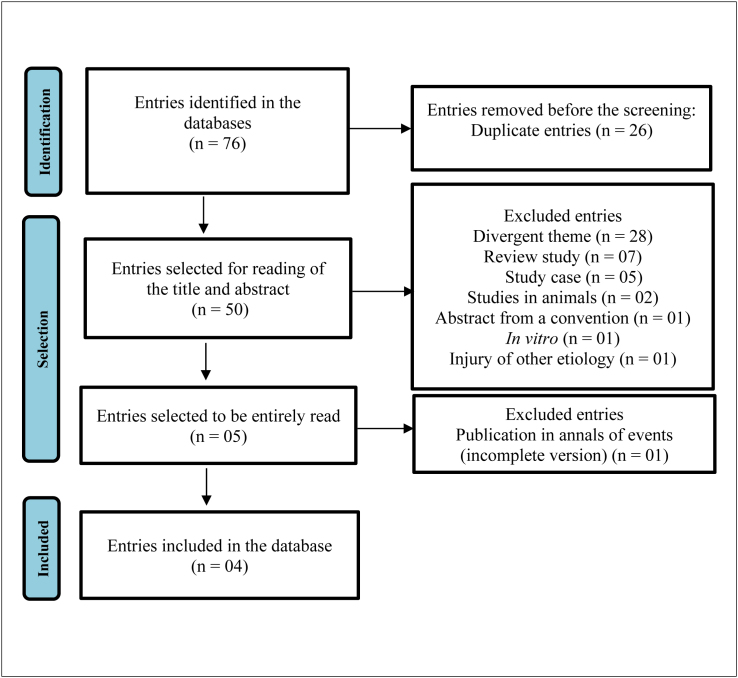
Flow chart depicting the study selection using Preferred Reporting Items for Systematic Reviews and Meta-Analyses (PRISMA) 2020 guidelines.

**Table 2:** Quantitative evaluation of the methodological quality

Authors / Article	Critical analysis of the Methodological Quality													Total Yes
	Questions													
	Q1	Q2	Q3	Q4	Q5	Q6	Q7	Q8	Q9	Q10	Q11	Q12	Q13	
Carrinho et al.^21^– A1	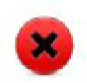	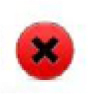	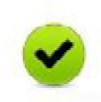	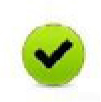	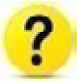	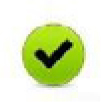	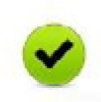	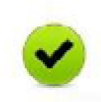	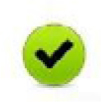	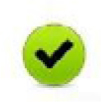	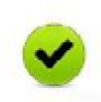	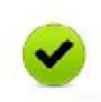	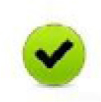	11
Tardivo et al.^22^– A2	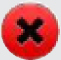	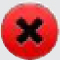	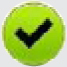	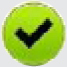	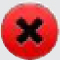	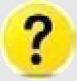	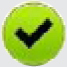	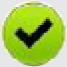	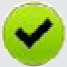	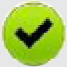	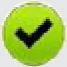	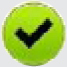	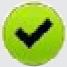	09
Mannucci et al.^23^– A3	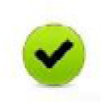	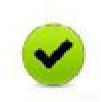	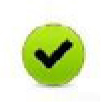	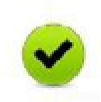	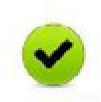	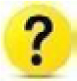	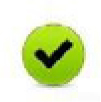	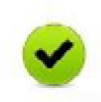	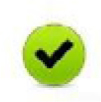	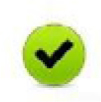	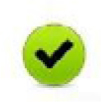	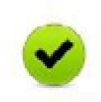	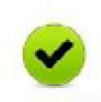	11
Morley et al.^24^– A4	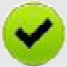	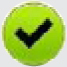	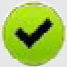	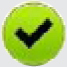	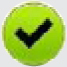	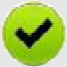	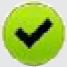	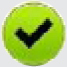	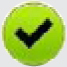	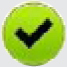	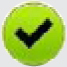	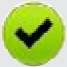	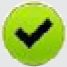	13

(Yes) 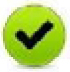

(No) 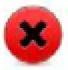

(Unclear) 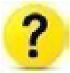

**Graphic 1:**
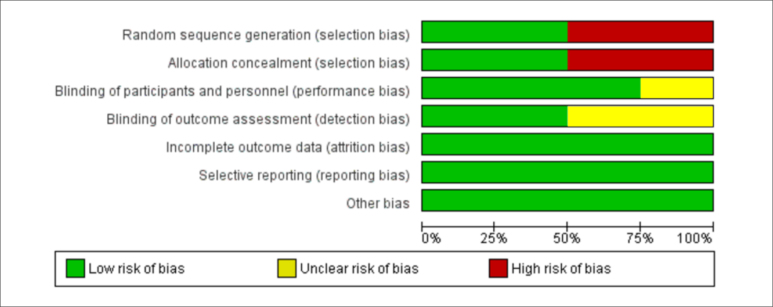
Distribution of risk of bias.

### Participants

The participants were between 18 and 35 years of age with a confirmed diagnosis of DM and at least one foot ulcer.

### Treatment groups

In all studies, PDT was only used in the experimental groups, whereas the control groups were treated with collagenase and chloramphenicol;^21^ systemic antibiotics and simple dry bandages;^22^ oral antibiotics and gauze embedded in vaseline;^23^ or an absorbent cover (Table 3).^24^

**Table 3:** Description of the studies included in the systematic review

Article	Local	Study design Evidence level	Population	Groups	Outcome measurement	Main results
A1	Brazil	Non-randomized clinical trial controlled with placebo 1.d	12	Experimental group Sample: 06 Intervention: PDT protocol and primary cover with collagenase 0.6 U/g + chloramphenicol 0.01 g/g. Control group Sample: 06 Intervention: primary cover with collagenase 0.6 U/g + chloramphenicol 0.01 g/g.	– Nominal analysis of the ulcers – Healing index of the ulcer	– There was an improvement in tissue repair in the patients treated with PDT, especially in the macroscopic aspects of neovascularization, epithelialization, and reduction of the wound’s affected area. – A significant difference in the nominal size of the wounds pre- and post-treatment with PDT was observed along with the wound’s affected area (P = 0.036).
A2	Brazil	Non-randomized clinical trial controlled with placebo 1.d	34	Experimental group Sample: 18 Intervention: PDT protocol, systemic antibiotics (clindamycin 300 mg every 8 hours and ciprofloxacin 500 mg every 12 hours) for 10 days and simple dry bandages. Control group Sample: 16 Intervention: systemic antibiotics (clindamycin 300 mg every 8 hours and ciprofloxacin 500 mg every 12 hours) for 10 days and simple dry bandages.	– Photographic analysis – Simple radiographies of the bones from the feet – Wagner’s classification	– Only one of the patients from the experimental group required amputation. At least two participants in the PDT group recovered from resistant bacterial strains (*Pseudomonas aeruginosa* and *Klebsiella pneumoniae*). – In the control group, every participant underwent amputation. – The amputation rate in the PDT group was 0.029 times the rate of the control group (P = 0.002), with an amputation rate 35 times lesser in the former than that in the latter.
A3	Italy	Non-randomized clinical trial controlled with placebo 1.c	55	Experimental group Sample: 42 Intervention: PDT protocol, 875 mg of oral Amoxicillin + clavulanic acid 125 mg, three times per day, for 7 days and cover with gauze embedded in Vaseline. Control group Sample: 13 Intervention: 875 mg of oral Amoxicillin + clavulanic acid 125 mg, three times per day, for 7 days and cover with gauze embedded in Vaseline.	– Microbiological analysis – Nominal analysis of the ulcers – PEDIS score	– A reduction in the total microbial load immediately after illumination, with a progressive fading of the effect during follow-up. – No significant changes were observed in the ulcer’s dimensions following PDT.
A4	United Kingdom	Non-randomized clinical trial controlled with placebo 1.c	16	Experimental group Sample: 08 Intervention: PDT protocol and cover with absorption action. Control group Sample: 08 Intervention: cover with absorption action.	– Microbiological analysis – Nominal analysis of the ulcers	– The treatment was well-tolerated without reports of pain. – There was a significant statistical reduction (P < 0.001) in the bacterial load immediately after PDT; in contrast, there was no significant reduction in the placebo group. – There was a better healing process—measured using the wound size—in patients who received PDT in comparison with those in the control group.

PDT = photodynamic therapy.

### Outcomes

Two studies^22,24^ reported reduced microbial load as the primary outcome and changes in tissue repair as the secondary outcome. Carrinho et al.^21^ evaluated the changes in tissue repair as the primary outcome. Tardivo et al.^22^ evaluated foot amputation as the primary outcome and changes in tissue repair as the secondary outcome. To measure the effects of PDT on the microbial load, microbiological analyses of ulcer swabs were performed.^23,24^ Healing was evaluated using photographs,^22^ assessments of the area affected,^21,23^ and perfusion, extent, depth, infection and sensation (PEDIS) score.^23^ Radiological assessments and Wagner's classification analysis were used to identify the need for amputation.^22^

### Main results of PDT in the treatment of foot ulcers

PDT resulted in significant improvements in microbial load and tissue repair compared to the interventions in the control groups. The clinical evolution of the ulcer also demonstrated a significant improvement (P = 0.036) with the use of PDT.^21^ PDT also promoted improvements in infection control (P < 0.01).^24^ PDT was well-tolerated when compared with the interventions in the control groups.^23^ The amputation rate was 35 times lower in the group that received PDT in comparison with the groups that received other treatments (P = 0.002).^22^ The most common photosensitizer was methylene blue (0.01% to 1%). The wavelength ranged from 560 nm to 689 nm with doses of 6–30 J/cm^2^. The number of PDT sessions varied from one to 23. The longest follow-up post-PDT was 90 days (Table 4).

**Table 4:** Description of the main parameters of the photodynamic therapy (PDT) protocols

Article	Photosensitive composite	Concentration / Rest period	Wavelength	Dose (J)	Application period	Total number of sessions	Frequency of applications	Follow-up period
A1	Methylene blue	0.01% 5 minutes	660 nm	6 J/cm^2^	8 seconds per cm^2^	10 sessions	3 times a week	22 days
A2	Methylene blue and tolonium chloride	1% Did not report	560 and 640 nm	6 J/cm^2^and 30 J/cm^2^	10 minutes in every area	Average of 16 sessions (9–23)	2 times a week	Did not report
A3	Methylene blue	0.10, 0.30, and 0.50% 60 minutes	689 ± 5 nm	60 J/cm^2^	8 minutes and 30 cm^2^	Unique application	-	15 days
A4	Methylene blue	Did not report 15 minutes	570–670 nm	50 J/cm^-2^ (Total)	Did not report	Unique application	-	90 days

### Quality of evidence

The evidence regarding the outcomes concerning the reduction in the microbial load, improvements in tissue repair, and reduction in amputations was of “moderate quality,” and the outcomes were considered clinically critical (essentials) for the patients. Therefore, there is moderate confidence in the estimated effect but more clinical trials can improve the confidence (Table 5).

**Table 5:** Quality of evidence of the outcomes

EVALUATION OF THE QUALITY OF OUTCOMES								
Number of studies	Study design	Risk of bias	Inconsistence	Indirect evidence	Imprecision	Other considerations	Quality of the evidence	Importance
**Outcome 1 – Reduction in the microbial load**								
02	Clinical trials	not serious	not serious	not serious	serious^a^	none	⨁⨁⨁◯MODERATE	CRITICAL
**Outcome 2 – Progress in tissue repair**								
04	Clinical trials	serious^b^	not serious	not serious	not serious	none	⨁⨁⨁◯ MODERATE	CRITICAL
Outcome 3 – Reduction in amputations due to diabetic foot								
01	Clinical trials	serious^b^	not serious	not serious	not serious	none	⨁⨁⨁◯ MODERATE	CRITICAL

a Microbial load assessment using the swab technique (inaccurate for diabetic foot ulcers [DFU] microbial assessment);

b No real randomization (blinding of intervention applicators).

### Effectiveness of PDT in the treatment of foot ulcers with infections

Numerous variabilities were noted in terms of the control groups and the methods of analysis of the evaluated outcomes between the studies. The studies used different parameters in their evaluations, which resulted in high heterogeneity (I^2^ = 99%). Therefore, the outcomes of tissue repair were verified in this meta-analysis. However, nested meta-analyses were not performed. The effect of PDT on tissue repair was significant, thus suggesting the benefits of using PDT protocols in DFU. There were significant differences between the treatments (PDT protocol and standard therapy), and the intervention favored the experimental group (P = 0.04) (Graphic 2).

**Graphic 2:**
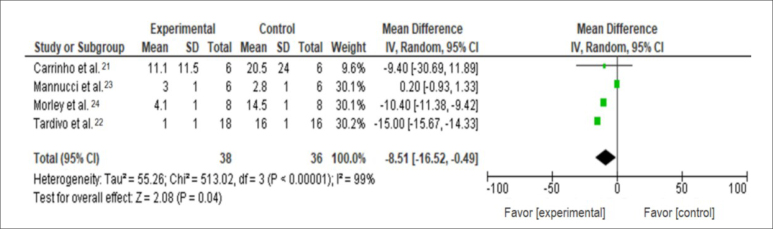
Meta-analysis regarding the outcome of tissue repair.

## DISCUSSION

There is a consensus regarding the superiority of PDT treatment for DFU over other therapies in control groups. This finding may be related to PDT's capacity to induce cell death in pathogens, decrease inflammation, and stimulate the proliferation of fibroblasts, collagen, and elastin.^25,26^ Owing to its mechanism of action, low invasiveness, and absence of significant collateral effects, PDT offers an alternative treatment for DFU.^27^

In the studies evaluated, patients treated with PDT demonstrated a significant reduction in the microbial load.^23,24^ Other studies have corroborated this result.^12,28,29^ An *in vitro* study demonstrated that PDT exerted bactericidal effects on resistant bacteria and biofilms through oxidative stress.^10^ This is a relevant result as biofilm bacteria and deep DFU cultures have broad bacterial resistance.^30,31^ It is worth highlighting that increasing antimicrobial resistance has restricted the therapeutic arsenal to face this type of infection,^32^ which reinforces the need for new treatments with antimicrobial action and reduce the clinical indications for antibiotics.^33^

Several studies have confirmed that PDT is a promising adjuvant approach in the deactivation of resistant bacteria and bacterial biofilms.^10,25,34–36^ In two studies, bacterial colonies were decreased shortly after the first PDT session.^23,24^ This finding is a relevant benefit since a decrease in the colonies that form the biofilm around the ulcer provides a favorable environment for the formation of healthy granular tissue.^37^ Consequently, this deactivation contributes to tissue repair.^12^ A better healing process of ulcers was noted with PDT.^21–24^

These findings demonstrate that PDT has several advantages in wound healing, especially in DFU.^1,11^ PDT is an adjuvant therapy that can substantially improve the healing process in DFU because it facilitates tissue repair through an immediate reduction in bacterial colonies.

In this study, we identified that through its antimicrobial effects and contribution to the healing process of infected ulcers, PDT can decrease the risk of amputation. Previous studies have reported similar results.^22,38^ This finding highlights PDT's relevance as an adjuvant therapy because it demonstrated the potential to minimize the risk of amputation and decrease treatment costs and hospitalizations.^33^

Despite the convergent and positive results of PDT, each study used varying parameters for photosensitizer activation. Consequently, it was not possible to determine the optimal parameters for PDT in the treatment of DFU. Further efforts have been made to standardize the PDT protocols.^39^ However, the most recent studies used lower doses for light irradiation.^21,22^ Reduction of the dose of light in the PDT protocol has been suggested to exert better biostimulatory effects in cells. Cells enriched with low amounts of photosensitizer composites may also proliferate better following light exposure with the correct wavelength and appropriate time window.^40^

No adverse events were associated with PDT. A recent study inferred that with the correct choice of PDT parameters, this approach is safe and reliable.^34^ For the three outcomes analyzed, the quality of evidence was considered moderate for an essential clinical outcome. In the meta-analysis, the intervention favored the experimental group. One study corroborated that PDT may be a promising procedure in the management of infected ulcers with a higher probability of healing, lower risk of amputation, and an important clinical outcome.^9^

However, it is pertinent to discuss the importance of health education with the patients during therapy, especially regarding glycemic control, foot care, use of appropriate shoes, and a healthy lifestyle to improve the success of PDT.^13^

The current study contributes to improving the awareness of healthcare professionals regarding new protocols and medical therapies using adjuvant technologies with better cost-benefit relationships, such as PDT. Furthermore, PDT protocols validated by clinical trials that are identified in this review can be used by health professionals safely. Despite the limited research on PDT in DFU, the findings of this study highlight the relevance of antimicrobial therapy that can be used along with standard treatments to decrease bacterial resistance and non-traumatic amputations.

More clinical studies are needed with microbiological evidence of the effectiveness of PDT in decreasing the microbial load as well as identifying the most effective PDT parameters in the treatment of DFU. This is essential for determining the precise mechanisms of action of PDT and the interactions between light and injured tissues as well as selecting the appropriate LASER parameters and concentration of the photosensitizer to avoid thermal discomfort in the irradiated tissues or phototoxicity.

## CONCLUSION

PDT is significantly more effective in treating infected DFU than standard care. A significant reduction in the infection rate was noted soon after the first session of PDT along with an improvement in healing. The three outcomes analyzed were supported by a moderate quality of evidence and essential clinical outcomes. In the meta-analysis, the intervention significantly favored the groups treated with PDT.
